# Mucosal antibody responses following Vaxzevria vaccination

**DOI:** 10.1111/imcb.12685

**Published:** 2023-09-05

**Authors:** Kevin J Selva, Pradhipa Ramanathan, Ebene R Haycroft, Chee Wah Tan, Lin‐Fa Wang, Laura E Downie, Samantha K Davis, Ruth A Purcell, Helen E Kent, Jennifer A Juno, Adam K Wheatley, Miles P Davenport, Stephen J Kent, Amy W Chung

**Affiliations:** ^1^ Department of Microbiology and Immunology, Peter Doherty Institute for Infection and Immunity University of Melbourne Melbourne VIC Australia; ^2^ Programme in Emerging Infectious Diseases Duke‐NUS Medical School Singapore; ^3^ Infectious Diseases Translational Research Programme, Department of Microbiology and Immunology, Yong Loo Lin School of Medicine National University of Singapore Singapore; ^4^ Singhealth Duke‐NUS Global Health Institute Singapore; ^5^ Department of Optometry and Vision Sciences University of Melbourne Carlton VIC Australia; ^6^ Kirby Institute, University of New South Wales Kensington NSW Australia; ^7^ Melbourne Sexual Health Centre and Department of Infectious Diseases Alfred Hospital and Central Clinical School, Monash University Melbourne VIC Australia

**Keywords:** antibodies, COVID‐19, saliva, SARS‐CoV‐2, tears, Vaxzevria

## Abstract

Mucosal antibodies play a key role in protection against breakthrough COVID‐19 infections and emerging viral variants. Intramuscular adenovirus‐based vaccination (Vaxzevria) only weakly induces nasal IgG and IgA responses, unless vaccinees have been previously infected. However, little is known about how Vaxzevria vaccination impacts the ability of mucosal antibodies to induce Fc responses, particularly against SARS‐CoV‐2 variants of concern (VoCs). Here, we profiled paired mucosal (saliva, tears) and plasma antibodies from COVID‐19 vaccinated only vaccinees (uninfected, vaccinated) and COVID‐19 recovered vaccinees (COVID‐19 recovered, vaccinated) who both received Vaxzevria vaccines. SARS‐CoV‐2 ancestral‐specific IgG antibodies capable of engaging FcγR3a were significantly higher in the mucosal samples of COVID‐19 recovered Vaxzevria vaccinees in comparison with vaccinated only vaccinees. However, when IgG and FcγR3a engaging antibodies were tested against a panel of SARS‐CoV‐2 VoCs, the responses were ancestral‐centric with weaker recognition of Omicron strains observed. In contrast, salivary IgA, but not plasma IgA, from Vaxzevria vaccinees displayed broad cross‐reactivity across all SARS‐CoV‐2 VoCs tested. Our data highlight that while intramuscular Vaxzevria vaccination can enhance mucosal antibodies responses in COVID‐19 recovered vaccinees, restrictions by ancestral‐centric bias may have implications for COVID‐19 protection. However, highly cross‐reactive mucosal IgA could be key in addressing these gaps in mucosal immunity and may be an important focus of future SARS‐CoV‐2 vaccine development.

## Graphical abstract

COVID‐19 recovered individuals vaccinated with Vaxzevria (AstraZeneca) induced higher levels of salivary IgA that display reactivity across SARS‐CoV‐2 variants of concern including Omicron. In comparison, IgG and FcγR3a engaging antibodies responses were ancestral‐centric with weaker recognition of Omicron strains observed.

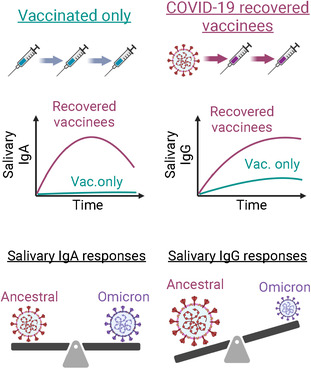

## INTRODUCTION

Mucosal immunity, particularly SARS‐CoV‐2 spike‐specific mucosal IgA, has been suggested to be protective against breakthrough COVID‐19 infections.^1^, ^2^ Unfortunately, intramuscularly (IM) administered COVID‐19 vaccines do not induce optimal mucosal antibody responses in uninfected COVID‐19 vaccinees.^3^ Vaxzevria is an IM chimpanzee adenoviral vector COVID‐19 vaccine used globally and is effective in reducing severe disease and death after two primary doses.^4^, ^5^

Recently, it has been shown that Vaxzevria induces better mucosal IgG and IgA responses in the nasal fluid of COVID‐19 recovered vaccinees than in vaccinated only vaccinees.^6^ In addition, while SARS‐CoV‐2 variants of concern (VoC) may escape neutralization, non‐neutralizing antibodies remain robust in inducing Fc effector functions – which have been suggested to contribute to protective immunity.^7^, ^8^ Nonetheless, little is known about how prior COVID‐19 infection impacts Fc‐receptor engagement by mucosal antibodies, particularly against the SARS‐CoV‐2 VoCs in individuals receiving Vaxzevria vaccines. Here, we compare antibody isotypes, subclasses, as well as Fc‐receptor engagement and cross‐reactivity across VoCs in mucosal secretions (saliva, tears) with plasma responses from Vaxzevria vaccinees.

## RESULTS

### Fc‐engagement by mucosal antibodies is enhanced in COVID‐19 recovered vaccinees

**Figure 1:**
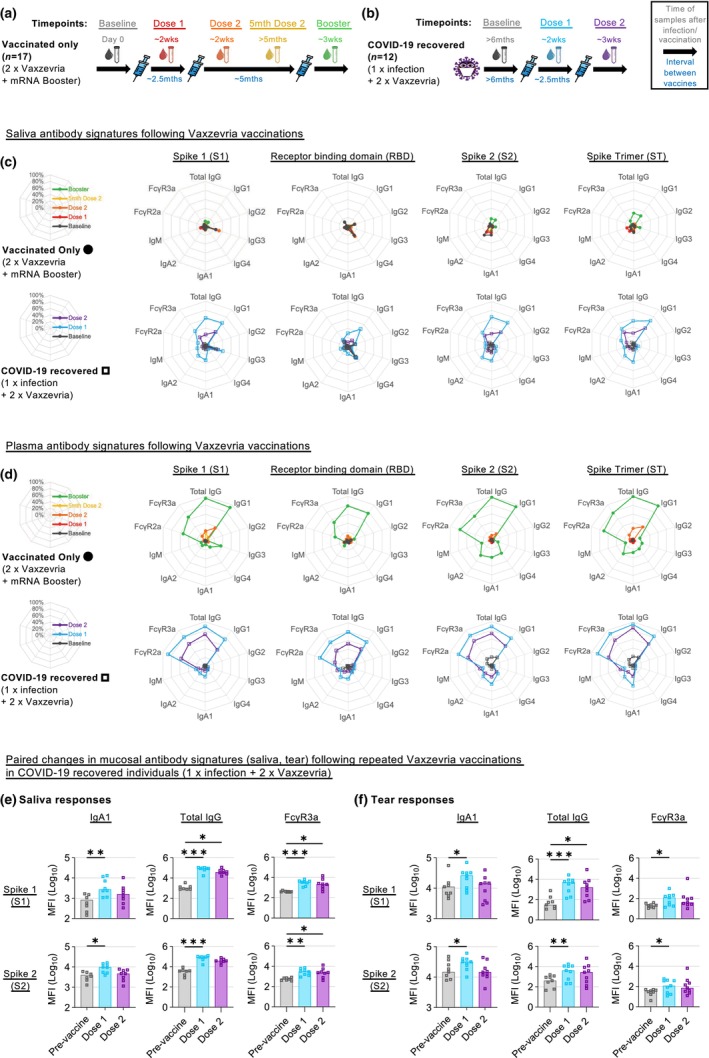
COVID‐19 recovered Vaxzevria vaccinees displayed enhanced IgA and FcγR responses in saliva and tear fluid. Paired saliva and plasma samples were collected pre‐ and post‐ Vaxzevria vaccination from vaccinated only **(a)** and COVID‐19 recovered **(b)** individuals at the indicated time‐points. Saliva **(c)** and plasma **(d)** antibody isotype and subclass responses from both cohorts against the various SARS‐CoV‐2 spike antigens were compiled into respective radar plots. To transform the data into percentages for use in the radar plots **(c, d)**, the median of each cohort/timepoint's antigen‐specific MFI was divided by the antigen‐specific MFI in the 99th percentile for that detector (99th percentile was chosen to minimize the impact of outliers on the data transformation). IgA, total IgG and FcγR3a saliva **(e)** and tear **(f)** antibody features from COVID‐19 recovered individuals after their second Vaxzevria vaccination are also illustrated in respective bar graphs. Statistical significance was calculated using the Friedman test followed by Dunn's test for multiple comparisons and where significant or trending significance, *P*‐values are reported (*P ≤ 0.05; **P ≤ 0.01; ***P ≤ 0.001; ****P ≤ 0.0001).

To investigate how prior COVID‐19 infection impacts humoral responses following Vaxzevria vaccination, we compared both COVID‐19 vaccinated only vaccinees (vaccinated only; up to 2 × Vaxzevria + 1 × mRNA booster; Figure 1a) and COVID‐19 recovered vaccinees (COVID‐19 recovered; 1 × prior COVID‐19 infection + 2 × Vaxzevria; Figure 1b; Table 1). As Vaxzevria encodes the ancestral SARS‐CoV‐2 Spike, we first profiled antibody signatures made against the different regions of the ancestral spike, namely the receptor binding domain (RBD), Spike 1 (S1), Spike 2 (S2), and the whole Spike trimer (ST).

**Table 1:** Summary of Vaxzevria vaccinated cohorts.

Variable	Vaxzevria vaccinees		
	Vaccinated only (*n* = 17)	COVID‐19 recovered (Convalescent, vaccinated) (*n* = 12)
Age, mean (range), years	46.5 (23–69)	61.2 (51–72)
Gender		
Female (%)	10 (58.8)	4 (33.3)
Male (%)	7 (41.2)	8 (66.7)
Time from symptom onset until sample collection, mean (range), days		414 (262–458)
Vaccination		
Time after 1st shot until sample collection, mean (range), days	13.4 (7–22)	12.7 (8–16)
Time between 1st and 2nd shot, mean (range), days	83 (70–92)	81 (70–86)
Time after 2nd shot until sample collection, mean (range), days	13.4 (7–22)	17.6 (12–26)
Time between 2nd shot and 5 months sample collection, mean (range), days	149.6 (101–184)	
Time after 3rd shot until sample collection, mean (range), days	20.1 (10–31)	

Overall, COVID‐19 recovered individuals displayed elevated total IgG responses in both saliva and plasma after two antigen exposures (1 × prior COVID‐19 infection + 1 × Vaxzevria) compared with vaccinated‐only individuals (2 × Vaxzevria) (Figure 1c, d). These IgG responses were mainly composed of IgG1 across all four spike antigens tested (Supplementary figure 2a, c). Enhanced spike‐specific antibody engagements of Fc‐receptors, particularly FcγR3a (CD16), were also detected in saliva and plasma after two antigen exposures in COVID‐19 recovered individuals compared with vaccinated‐only individuals (Figure 1c, d; Supplementary figure 2a, c). Furthermore, significant increases in spike‐specific salivary IgA1 responses were detected in COVID‐19 recovered individuals following their first Vaxzevria vaccination (*P* < 0.05; Figure 1e).

In contrast, saliva and plasma responses against IgA1, total IgG as well as FcγR3a after COVID‐19 recovered individuals received their second Vaxzevria dose (1 × prior COVID‐19 infection + 2 × Vaxzevria) did not rise to the levels seen 2 weeks after the first dose (Figure 1c, d; Supplementary figure 2b, d). However, it should be noted that despite the decrease, total IgG and FcγR3a responses in saliva remained significantly elevated (*P* < 0.05) over pre‐vaccination responses particularly against the more novel Spike 1 protein (Figure 1e). Salivary IgA1 responses in COVID‐19 recovered individuals were comparable to pre‐vaccination levels after their second Vaxzevria dose. A similar trend was observed with IgA1, total IgG and FcγR3a responses in tear fluid from COVID‐19 recovered individuals after their Vaxzevria vaccination, with significant increases in antibody responses detected after the first Vaxzevria dose (*P* < 0.05; Figure 1f).

Compared with COVID‐19 recovered vaccinees, previously uninfected vaccinees displayed weak salivary and plasma antibody responses after their first and second Vaxzevria vaccines (2 × Vaxzevria) (Figure 1c, d; Supplementary figure 2a, c). However, plasma IgG and Fc‐engagement responses were greatly enhanced after Vaxzevria vaccinees received their mRNA booster (2 × Vaxzevria + 1 × mRNA booster) (Figure 1d, Supplementary figure 2d). In contrast, changes to salivary antibody responses were more modest after Vaxzevria vaccinees received their IM mRNA booster (2 × Vaxzevria +1 × mRNA booster) (Figure 1c). As such, Fc‐engagement by salivary antibodies remained significantly enhanced (*P* < 0.05) among COVID‐19 recovered vaccinees compared with vaccinated only vaccinees after three antigen exposures each, particularly against the more antigenically diverse RBD and Spike 1 (Supplementary figure 2b).

Taken together, our findings support previous observations that salivary IgA responses dip after the second IM vaccine dose in COVID‐19 recovered subjects, and suggest that repeated mucosal antigen exposures (either mucosal infection or potentially mucosal vaccination) may be required to maintain robust mucosal IgA and antibody‐mediated Fc‐responses.^9^

### Mucosal IgG and Fc‐antibodies target more ancestral‐centric SARS‐CoV‐2 antigens

**Figure 2:**
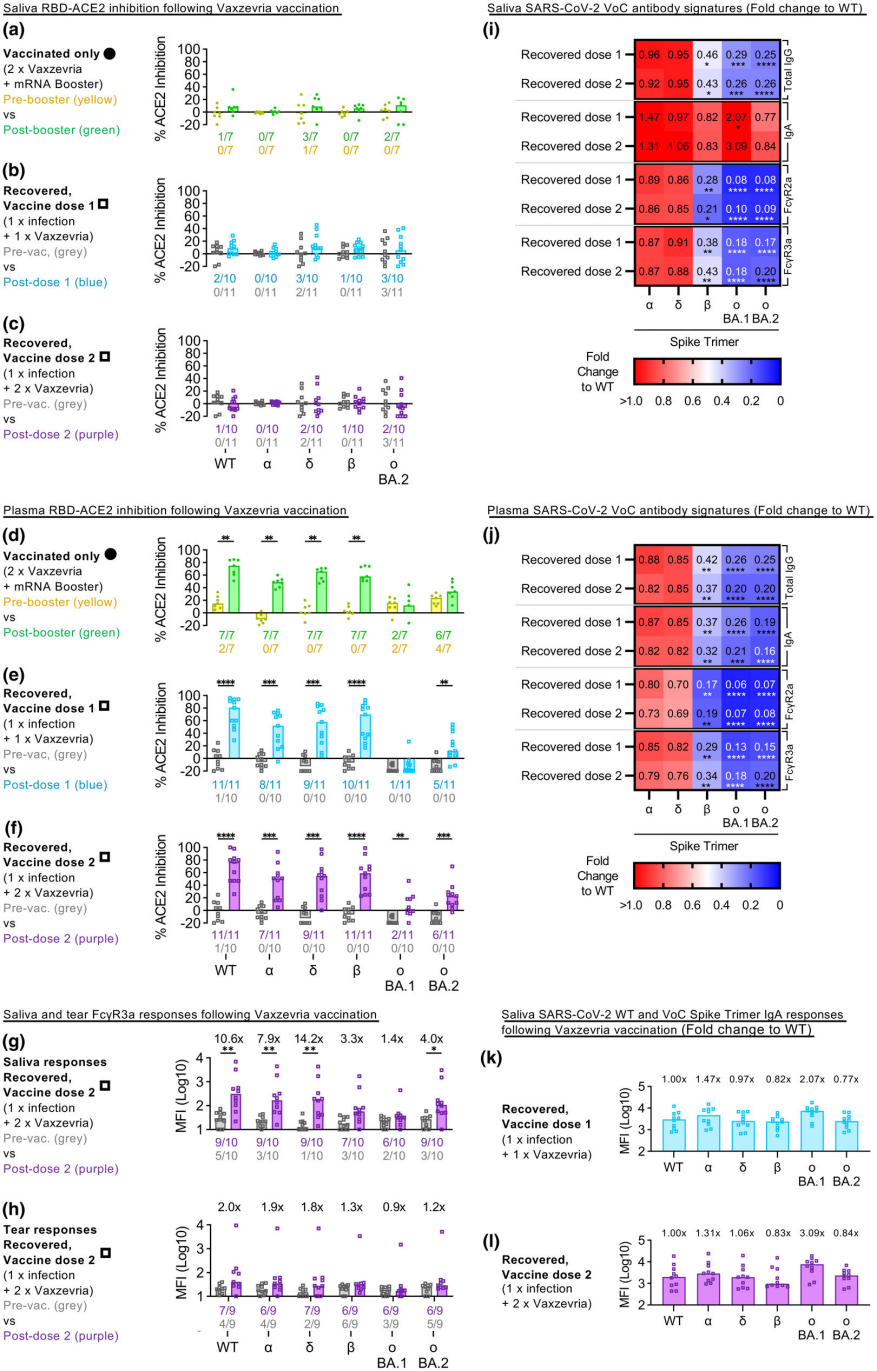
Cross‐reactive salivary IgA avoids vaccination‐induced ancestral‐centric bias. Bar graphs describe salivary **(a–c)** and plasma **(d–f)** inhibition of RBD‐ACE2 interactions against the ancestral wildtype (WT) SARS‐CoV‐2 or the VoCs (α, Alpha; δ, Delta; β, Beta; ο BA.1, Omicron BA.1; ο BA.2, Omicron BA.2) by vaccinated only **(a, d)** and COVID‐19 recovered individuals **(b, c, e, f)**, respectively. The number of individuals with detectable responses above the arbitrary 20% assay threshold (dotted line) at either timepoint are listed under the bar graphs in their respective colors. Significant differences between both timepoints were calculated using the two‐tailed Mann–Whitney *U*‐test, followed by Bonferroni‐Dunn's test for multiple comparisons. Bar graphs also show the salivary **(g)** and tear **(h)** FcγR3a responses against WT SARS‐CoV‐2 or the VoCs in COVID‐19 recovered individuals after their second Vaxzevria vaccination. Fold changes listed above the bar graphs were calculated for post dose 2 Vaxzevria responses (purple) over their respective pre‐vaccination responses (gray) for each antigen. The number of individuals with detectable responses above the assay threshold (median responses from COVID‐19 unvaccinated, uninfected healthy controls; dotted line) at either timepoint were listed under the bar graphs in their respective colors. Significant differences between both timepoints were calculated using the two‐tailed Mann–Whitney *U*‐test, followed by Bonferroni‐Dunn's test for multiple comparisons. Heat maps illustrate the VoC‐specific Spike Trimer salivary **(i)** and plasma **(j)** antibody responses post‐mRNA booster and post‐ Vaxzevria vaccination (dose 1 or 2) for both vaccinated only and COVID‐19 recovered cohorts respectively. The median antibody response for each VoC spike was described as a fold change to the wildtype spike. Statistical significance was calculated using Friedman's test followed by Dunn's test for multiple comparisons. Bar graphs show the salivary IgA responses against WT SARS‐CoV‐2 or the VoCs in COVID‐19 recovered individuals after their first **(k)** and second **(l)** Vaxzevria vaccines. Fold changes listed above are calculated against that for the WT SARS‐CoV‐2. WT SARS‐CoV‐2 salivary IgA responses from COVID‐19 unvaccinated and uninfected healthy controls are shown by the dotted line. Where significant, *P*‐values are reported (*P ≤ 0.05; **P ≤ 0.01; ***P ≤ 0.001; ****P ≤ 0.0001).

Mucosal neutralizing antibodies are key in preventing active SARS‐CoV‐2 infection and is the ongoing goal of nasal vaccine design.^10^, ^11^ Here, we explored whether IM Vaxzevria vaccines induce neutralizing activity at the mucosa, particularly among COVID‐19 recovered individuals, using a surrogate neutralization ACE2‐inhibition assay.^12^ We found only a few individuals with salivary antibody responses that could inhibit RBD‐ACE2 binding. Most individuals from both cohorts and after either one or two vaccines did not induce responses in saliva above the assay detection threshold (dotted line) (Figure 2a–c).

Vaccinated‐only individuals showed broad RBD‐ACE2 inhibitory activity in plasma against the SARS‐CoV‐2 VoCs after receiving their mRNA booster (2 × Vaxzevria + 1 × mRNA boost) (Figure 2d). Similarly, COVID‐19 recovered individuals showed broad RBD‐ACE2 inhibition in plasma against SARS‐CoV‐2 VoCs, even after their first Vaxzevria vaccine (Figure 2e, f).

Given the poor neutralizing activity in saliva following IM vaccination, mucosal Fc‐responses could play a larger role in protecting against active infections. As COVID‐19 recovered individuals induced better antibody‐mediated Fc‐engagement against the ancestral spike in their mucosal secretions, we next studied whether their mucosal antibodies could also induce effective Fc‐receptor engagement to the range of SARS‐CoV‐2 VoCs spikes. While we detected salivary antibody‐mediated Fc‐receptor engagement in COVID‐19 recovered vaccinees (1 × prior COVID‐19 infection + 2 × Vaxzevria) across a broad range of SARS‐CoV‐2 VoCs, these responses were ancestral‐centric with the largest responses against variants (Alpha, 7.9‐fold; Delta 14.2‐fold) more similar to the ancestral wildtype (Figure 2g). Whilst more modest, Fc‐engagement responses in tear fluid against the SARS‐CoV‐2 VoCs also showed a similar ancestral‐centric trend (Figure 2h).

### Mucosal IgA broadly recognizes SARS‐CoV variants

Immunological imprinting from repeated vaccinations with the ancestral spike may hamper the development of cross‐reactive humoral responses specific against novel SARS‐CoV‐2 variants such as Omicron.^13^, ^14^, ^15^ To investigate the impact of immunological imprinting on vaccine‐induced responses, we compared the antibody signatures against the VoCs against those made against the ancestral wildtype (Figure 2i, j; Supplementary figure 3a, b). Ancestral‐centric bias was largely detected for salivary and plasma total IgG and Fc‐engagement responses (*P* < 0.01), with weaker responses against the more novel Omicron variants BA.1 and BA.2 compared with variants more similar to the ancestral wildtype, such as Alpha and Delta (Figure 2i, j).

In contrast, salivary IgA, but not plasma IgA, showed cross‐reactivity across all the variants tested (Alpha, Delta, Beta, Omicron BA.1, Omicron BA.2) (Figure 2i–l; Supplementary figure 4a, b), particularly after the first Vaxzevria dose in COVID‐19 recovered individuals. As such, the induction and retention of localized cross‐reactive IgA at the mucosa could be vital in the protection against emerging SARS‐CoV‐2 variants. Larger cohort studies should be done to further explore these findings.

## DISCUSSION

Mucosal immunity is a key barrier in preventing active infections by respiratory pathogens, such as SARS‐CoV‐2. Higher levels of salivary antibodies, particularly secretory IgA targeting SARS‐CoV‐2 RBD, has been associated with protection against breakthrough infections.^2^ Our group and others have shown previously that IM COVID‐19 mRNA vaccinations are insufficient for inducing robust mucosal humoral responses in vaccinated only individuals.^3^, ^16^ Recently, it has been shown that COVID‐19 recovered individuals receiving IM Vaxzevria vaccination produced more IgG and IgA in their nasal secretions, compared with vaccinated only individuals.^6^

Here, we investigated whether COVID‐19 recovered individuals also generate improved IgG and IgA responses in other important mucosal secretions, namely saliva and tear fluid. Furthermore, we explored whether these mucosal antibodies induced after Vaxzevria vaccination could also effectively engage Fc‐receptors. Compared with vaccinated only individuals, COVID‐19 recovered individuals did induce better mucosal (saliva, tears) IgG, IgA1 and FcγR3a engagement responses after the first Vaxzevria dose. However, both systemic and mucosal antibody responses in COVID‐19 recovered individuals dipped after the second Vaxzevria vaccination.

Local antigen stimulation at the mucosa has been shown to be key to producing mucosal IgA.^11^, ^17^ As such, repeated IM vaccinations may be limited in the robust induction of mucosal IgA. Furthermore, antibody feedback from pre‐existing high‐affinity IgG antibodies has also been suggested to limit the humoral responses towards vaccination through epitope masking.^18^ Indeed, an extended dosing interval of 45 weeks between the first and second Vaxzevria dose has been shown to be four times more effective than a 12‐week interval.^19^ In our study, the dosing interval between the first and second Vaxzevria doses for COVID‐19 recovered individuals was only 70–86 days (10–12 weeks). This limited dosing interval may have influenced the humoral responses made after the second Vaxzevria dose. However, we also acknowledge that the week delay in our sampling process after the second Vaxzevria vaccination of COVID‐19 recovered individuals may have also contributed to the observed differences in antibodies responses.

Unsurprisingly, while we did detect neutralizing activity against SARS‐CoV‐2 in plasma following IM COVID‐19 vaccination, we did not detect robust salivary neutralizing activity even among COVID‐19 recovered vaccinees. This finding highlights the urgent need for an effective COVID‐19 mucosal vaccine as a tool to reduce breakthrough infections.^17^ In addition, while mucosal IgG antibodies could detect a broad range of SARS‐CoV‐2 VoCs and induce Fc‐receptor engagement, responses against novel variants such as Omicron were limited by ancestral‐centric bias. Immunological imprinting can not only influence mucosal humoral responses made after vaccination, but also responses following breakthrough infections.^15^ This emphasizes the need for future COVID‐19 vaccines to move away from the ancestral SARS‐CoV‐2 wildtype template and instead be updated with emerging variants.^20^

Finally, we observed that salivary IgA, but not plasma IgA, displayed cross‐reactivity across SARS‐CoV‐2 variants, particularly after the first Vaxzevria dose in COVID‐19 recovered individuals. Secretory IgA has been shown to be broadly cross‐reactive for different strains of influenza A and B viruses respectively.^21^, ^22^ Indeed, increased levels of ancestral SARS‐CoV‐2 wildtype spike‐specific mucosal IgA has been associated with a decreased risk of acquiring Omicron breakthrough infection.^1^ As such, the induction of cross‐reactive mucosal IgA could be key in preventing breakthrough infections by emerging SARS‐CoV‐2 variants. Given the modest size of our study, future studies involving larger cohorts of Vaxzevria vaccinees should further expand upon these findings.

Future work should focus on studying the effectiveness of updated mono‐ or bi‐valent COVID‐19 vaccines in reducing ancestral‐centric bias. The impact of repeated mucosal exposures, through either receiving mucosal vaccines or acquiring infections, on the levels of mucosal IgA and Fc‐responses could be further explored. Likewise, IgA and Fc‐responses in secretions from other mucosal sites where SARS‐CoV‐2 has been detected, such as the intestinal and nasal tracts, could also be investigated.

## CONCLUSION

IM Vaxzevria vaccination is not only effective in generating systemic humoral responses, but can also induce modest mucosal humoral responses, particularly among COVID‐19 recovered individuals. However, these IgG‐driven mucosal responses are largely non‐neutralizing and ancestral‐centric and may therefore have limited capacity to prevent breakthrough infection. Highly cross‐reactive mucosal IgA could be key in addressing these gaps in mucosal immunity and should be the emphasis of SARS‐CoV‐2 mucosal vaccines development.

## METHODS

### Cohort and sample collection

We enrolled individuals with (COVID‐19 positive between March to September 2020, thus likely infected with the ancestral virus or D614G strain) and without prior SARS‐CoV‐2 infection from a previously described cohort^23^ to donate blood, saliva, and tear samples prior to and following vaccinations with Vaxzevria (Oxford‐AstraZeneca) vaccines, as well as mRNA boosters (Table 1; Supplementary table 1). Whole blood was collected with sodium heparin anticoagulant (Becton Dickinson, Franklin Lakes, NJ USA) and plasma was collected and stored at −80°C until use. Saliva was collected by SalivaBio Oral Swabs (Salimetrics, Carlsbad, CA, USA) and processed following the manufacturer's instructions, before being stored at −80°C until use. Basal (non‐stimulated) tear samples (∼7 μL per eye) were collected by capillary flow (Drummond Scientific, Broomall, PA, USA) from the inferior tear meniscus as previously reported, and also stored at −80°C until use.^24^ Plasma and saliva from COVID‐19 unvaccinated, uninfected healthy controls were also collected on 16 March 2020, while tear controls were from pre‐pandemic samples. All participants provided written informed consent, and the study was approved by the University of Melbourne Human Research Ethics Committee (2021‐21198‐15398‐3, 2056689, 11507).

### Bead‐based multiplex against SARS‐CoV‐2 ancestral antigens

SARS‐CoV‐2 specific antibody isotypes (IgG, IgA, IgM) and subclasses (IgG1‐4, IgA1‐2) in plasma (1:1600), saliva (1:12.5) and tear (1:25) from pre‐pandemic and vaccinated cohorts were assessed using a customized multiplex bead‐based array consisting of four ancestral SARs‐CoV‐2 proteins, including Spike 1 (S1; Sino Biological, Beijing, China), Spike 2 (S2; Acro Biosystems, Newark, NJ, USA), Receptor Binding Domain (RBD) and whole Spike Trimer (ST), as described previously.^3^ SIVgp120 protein (Sino Biological) and uncoupled BSA‐blocked beads were included as negative controls for background subtraction. Plasma and saliva concentrations used in the array were chosen based on a dilution series. Briefly, antigen‐coupled beads were incubated with the respective samples on a shaker overnight at 4°C, before being washed, and incubated with Phycoerythrin (PE)‐conjugated detection antibodies (Southern Biotech, Birmingham, AL, USA) on a shaker for 2 h at room temperature (RT). The beads were then washed and read on the Flexmap 3D. Assays were repeated in duplicate (Supplementary figure 1a, b).

Engagement of SARS‐CoV‐2 specific antibodies to Fc gamma receptors (FcγR) were measured using surrogate Fc gamma receptor dimers (FcγR2a, CD32; FcγR3a, CD16) as described previously (kind gift from Mark Hogarth and Bruce Wines).^25^ After incubation with samples, the washed beads were first incubated with surrogate FcγR dimers on a shaker for 2 h at RT, washed again, and then incubated with Streptavidin‐R‐Phycoerythrin (SAPE; Thermo Fisher Scientific, Waltham, MA, USA) on a shaker for a further 2 h at RT. Finally, the beads were washed and read on the Flexmap 3D. Assays were repeated in duplicate.

### Bead‐based multiplex against SARS‐CoV‐2 variant antigens

To compare plasma (1:25600), saliva (1:12.5) and tear (1:25) SARS‐CoV‐2 ancestral WT and variant antibody responses (Alpha, Beta, Delta, Omicron BA.1, Omicron BA.2), WT and VoC Spikes were used to form a customized bead array (Supplementary figure 3a, b). To measure SARS‐CoV‐2 specific total IgG and IgA responses, beads were first incubated with samples, washed and then incubated with the biotin‐conjugated detection antibodies (MabTech, Nacka Strand, Sweden) on a shaker for 2 h at RT. Subsequently, the beads were washed and then incubated with SAPE for another 2 h at RT. Finally, the beads were washed again and then read on the Flexmap 3D. The ability of SARS‐CoV‐2 variant specific plasma (1:12800) and saliva (1:12.5) antibodies to mediate FcγR engagements (FcγR2a, CD32; FcγR3a, CD16) were measured using the surrogate Fc‐receptor dimers as described above. Assays were repeated in duplicate.

### Surrogate RBD‐ACE‐2 inhibition assay

Neutralizing activity of plasma (1:800, 1:4000) and saliva (1:12.5) against the SARS‐CoV‐2 variants of concern (Alpha, Beta, Delta, Omicron BA.1, Omicron BA.2) (Sino biological) were accessed using a surrogate RBD‐ACE‐2 inhibition assay. As described previously,^3^, ^12^ ancestral or variant RBD‐coupled beads were incubated with avi‐tagged biotinylated ACE2 in the presence of the respective plasma and saliva samples on a shaker for 2 h at RT. The beads were washed and then incubated with SAPE on a shaker for 1 h at RT. R‐Phycoerythrin Biotin‐XX conjugate (Thermo Fisher Scientific) was then added to the beads and incubated on a shaker for a further hour at RT. Finally, the beads were washed and read on the Flexmap 3D. Assays were repeated in duplicate. Calculation of % ACE2 inhibition was done using the following formula: ([1 – (Average MFI of sample wells/Average MFI of ACE2 only wells)] × 100). Graphs were plotted between −20% to 100% ACE2 inhibition. A nominal cutoff of 20% (depicted by dotted line) was also set as described previously^12^ (Supplementary figure 1c, d).

To validate that the RBD‐ACE2 inhibition assay could detect ACE2 inhibition in the presence of saliva, we spiked dilutions of either plasma from an mRNA boosted individual (1:200–1:25 600 final plasma dilutions) or a RBD neutralizing antibody SAD‐S35 (40–0.002 nM) in saliva collected from three COVID‐19 unvaccinated, uninfected healthy individuals (Supplementary figure 1e, f).

### Data transformation and statistical analysis

Statistical analysis was performed with GraphPad Prism 9 (GraphPad Software). Prior to analysis of the multiplex data (ancestral and variant antigens), the readings from negative control antigens (SIV gp120, BSA) were background subtracted for each individual antigen/detector. The multiplex data were then right shifted to remove negative values. To transform the multiplex data (ancestral antigens) into percentages for use in the radar plots (Figure 1c, d), the median of each cohort/timepoint's antigen‐specific MFI was divided by the antigen‐specific MFI in the 99th percentile for that detector (99th percentile was chosen to minimize the impact of outliers on the data transformation). Antibody levels between cohort/timepoints were compared using Mann–Whitney *U*‐tests or Friedman tests, with corrections for multiple comparisons as required.

## AUTHOR CONTRIBUTIONS

**Kevin J Selva:** Conceptualization; data curation; formal analysis; investigation; methodology; writing – original draft. **Pradhipa Ramanathan:** Investigation; methodology. **Ebene R Haycroft:** Investigation; methodology. **Chee Wah Tan:** Investigation. **Lin‐Fa Wang:** Investigation. **Laura E Downie:** Investigation. **Samantha K Davis:** Investigation. **Ruth A Purcell:** Investigation. **Helen E Kent:** Investigation. **Jennifer A Juno:** Investigation. **Adam K Wheatley:** Investigation. **Miles P Davenport:** Investigation; writing – review and editing. **Stephen J Kent:** Data curation; formal analysis; funding acquisition; investigation; methodology; project administration; writing – review and editing. **Amy W Chung:** Conceptualization; data curation; formal analysis; funding acquisition; investigation; methodology; supervision; writing – review and editing.

## CONFLICT OF INTEREST

The authors declare no conflict of interest.

## Supporting information


Supplementary figure 1.
Supplementary figure 2.
Supplementary figure 3.
Supplementary figure 4.
Supplementary table 1.

## Data Availability

The data that support the findings of this study are available on request from the corresponding author.

## References

[1] Havervall S , Marking U , Svensson J , *et al*. Anti‐spike mucosal IgA protection against SARS‐CoV‐2 omicron infection. N Engl J Med 2022; 387: 1333–1336. 36103621 10.1056/NEJMc2209651 PMC9511632

[2] Zuo F , Marcotte H , Hammarstrom L , Pan‐Hammarstrom Q . Mucosal IgA against SARS‐CoV‐2 omicron infection. N Engl J Med 2022; 387: e55. 10.1056/NEJMc2213153 36416778

[3] Selva KJ , Davis SK , Haycroft ER , *et al*. Tear antibodies to SARS‐CoV‐2: implications for transmission. Clin Transl Immunology 2021; 10: e1354. 34754451 10.1002/cti2.1354 PMC8559894

[4] Menni C , May A , Polidori L , *et al*. COVID‐19 vaccine waning and effectiveness and side‐effects of boosters: a prospective community study from the ZOE COVID study. Lancet Infect Dis 2022; 22: 1002–1010. 35405090 10.1016/S1473-3099(22)00146-3 PMC8993156

[5] Katikireddi SV , Cerqueira‐Silva T , Vasileiou E , *et al*. Two‐dose ChAdOx1 nCoV‐19 vaccine protection against COVID‐19 hospital admissions and deaths over time: a retrospective, population‐based cohort study in Scotland and Brazil. Lancet 2022; 399: 25–35. 34942103 10.1016/S0140-6736(21)02754-9 PMC8687670

[6] Aksyuk AA , Bansal H , Wilkins D , *et al*. AZD1222‐induced nasal antibody responses are shaped by prior SARS‐CoV‐2 infection and correlate with virologic outcomes in breakthrough infection. Cell Rep Med 2023; 4: 100882. 36610390 10.1016/j.xcrm.2022.100882 PMC9750884

[7] Mackin SR , Desai P , Whitener BM , *et al*. Fc‐gammaR‐dependent antibody effector functions are required for vaccine‐mediated protection against antigen‐shifted variants of SARS‐CoV‐2. Nat Microbiol 2023; 8: 569–580. 37012355 10.1038/s41564-023-01359-1 PMC10797606

[8] Bartsch YC , Tong X , Kang J , *et al*. Omicron variant spike‐specific antibody binding and Fc activity are preserved in recipients of mRNA or inactivated COVID‐19 vaccines. Sci Transl Med 2022; 14: eabn9243. 35289637 10.1126/scitranslmed.abn9243 PMC8995028

[9] Sheikh‐Mohamed S , Isho B , Chao GYC , *et al*. Systemic and mucosal IgA responses are variably induced in response to SARS‐CoV‐2 mRNA vaccination and are associated with protection against subsequent infection. Mucosal Immunol 2022; 15: 799–808. 35468942 10.1038/s41385-022-00511-0 PMC9037584

[10] Zhou R , Wang P , Wong YC , *et al*. Nasal prevention of SARS‐CoV‐2 infection by intranasal influenza‐based boost vaccination in mouse models. EBioMedicine 2022; 75: 103762. 34942445 10.1016/j.ebiom.2021.103762 PMC8687884

[11] Mao T , Israelow B , Pena‐Hernandez MA , *et al*. Unadjuvanted intranasal spike vaccine elicits protective mucosal immunity against sarbecoviruses. Science 2022; 378: eabo2523. 36302057 10.1126/science.abo2523 PMC9798903

[12] Lopez E , Haycroft ER , Adair A , *et al*. Simultaneous evaluation of antibodies that inhibit SARS‐CoV‐2 variants via multiplex assay. JCI Insight 2021; 6: e150012. 34251356 10.1172/jci.insight.150012 PMC8409985

[13] Blom K , Marking U , Havervall S , *et al*. Immune responses after omicron infection in triple‐vaccinated health‐care workers with and without previous SARS‐CoV‐2 infection. Lancet Infect Dis 2022; 22: 943–945. 35691303 10.1016/S1473-3099(22)00362-0 PMC9183210

[14] Reynolds CJ , Pade C , Gibbons JM , *et al*. Immune boosting by B.1.1.529 (omicron) depends on previous SARS‐CoV‐2 exposure. Science 2022; 377: eabq1841. 35699621 10.1126/science.abq1841 PMC9210451

[15] Park YJ , Pinto D , Walls AC , *et al*. Imprinted antibody responses against SARS‐CoV‐2 omicron sublineages. Science 2022; 378: 619–627. 36264829 10.1126/science.adc9127 PMC12945441

[16] Nickel O , Rockstroh A , Wolf J , *et al*. Evaluation of the systemic and mucosal immune response induced by COVID‐19 and the BNT162b2 mRNA vaccine for SARS‐CoV‐2. PloS One 2022; 17: e0263861. 36256664 10.1371/journal.pone.0263861 PMC9578597

[17] Madhavan M , Ritchie AJ , Aboagye J , *et al*. Tolerability and immunogenicity of an intranasally‐administered adenovirus‐vectored COVID‐19 vaccine: an open‐label partially‐randomised ascending dose phase I trial. EBioMedicine 2022; 85: 104298. 36229342 10.1016/j.ebiom.2022.104298 PMC9550199

[18] Schaefer‐Babajew D , Wang Z , Muecksch F , *et al*. Antibody feedback regulates immune memory after SARS‐CoV‐2 mRNA vaccination. Nature 2023; 613: 735–742. 36473496 10.1038/s41586-022-05609-w PMC9876794

[19] Flaxman A , Marchevsky NG , Jenkin D , *et al*. Reactogenicity and immunogenicity after a late second dose or a third dose of ChAdOx1 nCoV‐19 in the UK: a substudy of two randomised controlled trials (COV001 and COV002). Lancet 2021; 398: 981–990. 34480858 10.1016/S0140-6736(21)01699-8 PMC8409975

[20] Hoffmann M , Behrens GMN , Arora P , *et al*. Effect of hybrid immunity and bivalent booster vaccination on omicron sublineage neutralisation. Lancet Infect Dis 2023; 23: 25–28. 36480944 10.1016/S1473-3099(22)00792-7 PMC9721839

[21] Okuya K , Yoshida R , Manzoor R , *et al*. Potential role of nonneutralizing IgA antibodies in cross‐protective immunity against influenza A viruses of multiple hemagglutinin subtypes. J Virol 2020; 94: e00408‐20. 32269119 10.1128/JVI.00408-20 PMC7307104

[22] Asahi‐Ozaki Y , Yoshikawa T , Iwakura Y , *et al*. Secretory IgA antibodies provide cross‐protection against infection with different strains of influenza B virus. J Med Virol 2004; 74: 328–335. 15332283 10.1002/jmv.20173

[23] Juno JA , Tan HX , Lee WS , *et al*. Humoral and circulating follicular helper T cell responses in recovered patients with COVID‐19. Nat Med 2020; 26: 1428–1434. 32661393 10.1038/s41591-020-0995-0

[24] Nguyen BN , Chung AW , Lopez E , *et al*. Meibomian gland dropout is associated with immunodeficiency at HIV diagnosis: implications for dry eye disease. Ocul Surf 2020; 18: 206–213. 32081622 10.1016/j.jtos.2020.02.003

[25] Selva KJ , van de Sandt CE , Lemke MM , *et al*. Systems serology detects functionally distinct coronavirus antibody features in children and elderly. Nat Commun 2021; 12: 2037. 33795692 10.1038/s41467-021-22236-7 PMC8016934

