# Supplementary material for: Mucosal antibody responses following Vaxzevria vaccination
Source: Immunol Cell Biol. 2023 Sep 5;101(10):975–83. doi: 10.1111/imcb.12685 (PMC10952200; doi:10.1111/imcb.12685)
Supplement: Supplementary file 1 — Supplementary figure 1. Supplementary figure 2. Supplementary figure 3. Supplementary figure 4. Supplementary table 1. [file IMCB-101-975-s001.pdf]

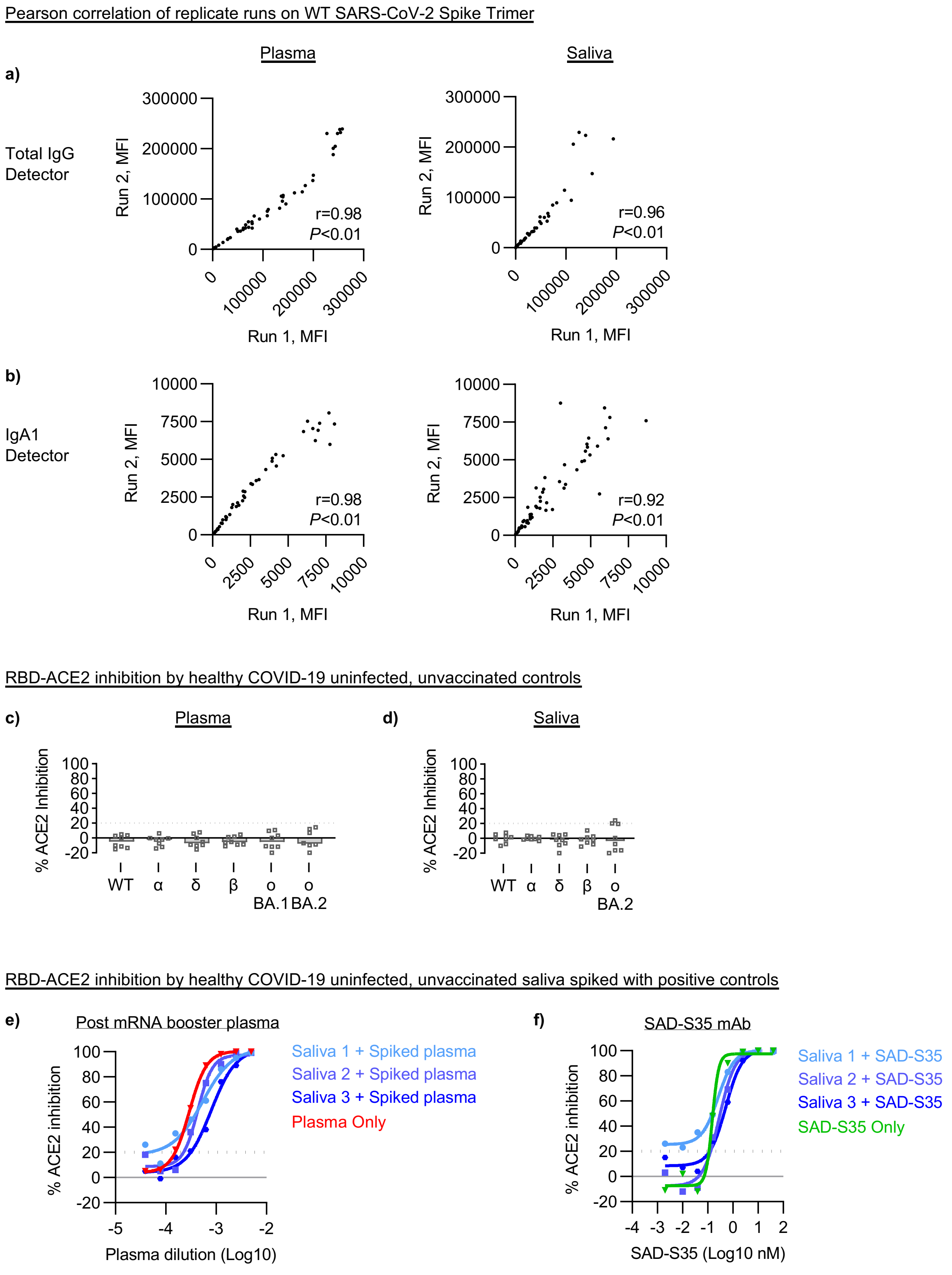

Supplementary figure 1: Controls for multiplex array and RBD-ACE2 inhibition assay.

Pearson correlations were performed comparing total IgG (a) and IgA1 (b) responses between replicate runs in both plasma and saliva. Bar graphs describe plasma (c) and salivary (d) inhibition of RBD-ACE2 interactions against the ancestral wildtype (WT) SARS-CoV-2 or the VoCs (α, Alpha; δ, Delta; β, Beta; o BA.2, Omicron BA.2) by healthy COVID-19 uninfected and unvaccinated controls. The assay threshold is displayed by a dotted line (arbitrary 20%). Line graphs describe inhibition of WT SARS-CoV-2 RBD-ACE2 interactions by post mRNA booster plasma (e) or RBD neutralizing antibody SAD-S35 (f) in the presence or absence of saliva from three healthy COVID-19 uninfected, unvaccinated individuals. The assay threshold is displayed by a dotted line (arbitrary 20%).

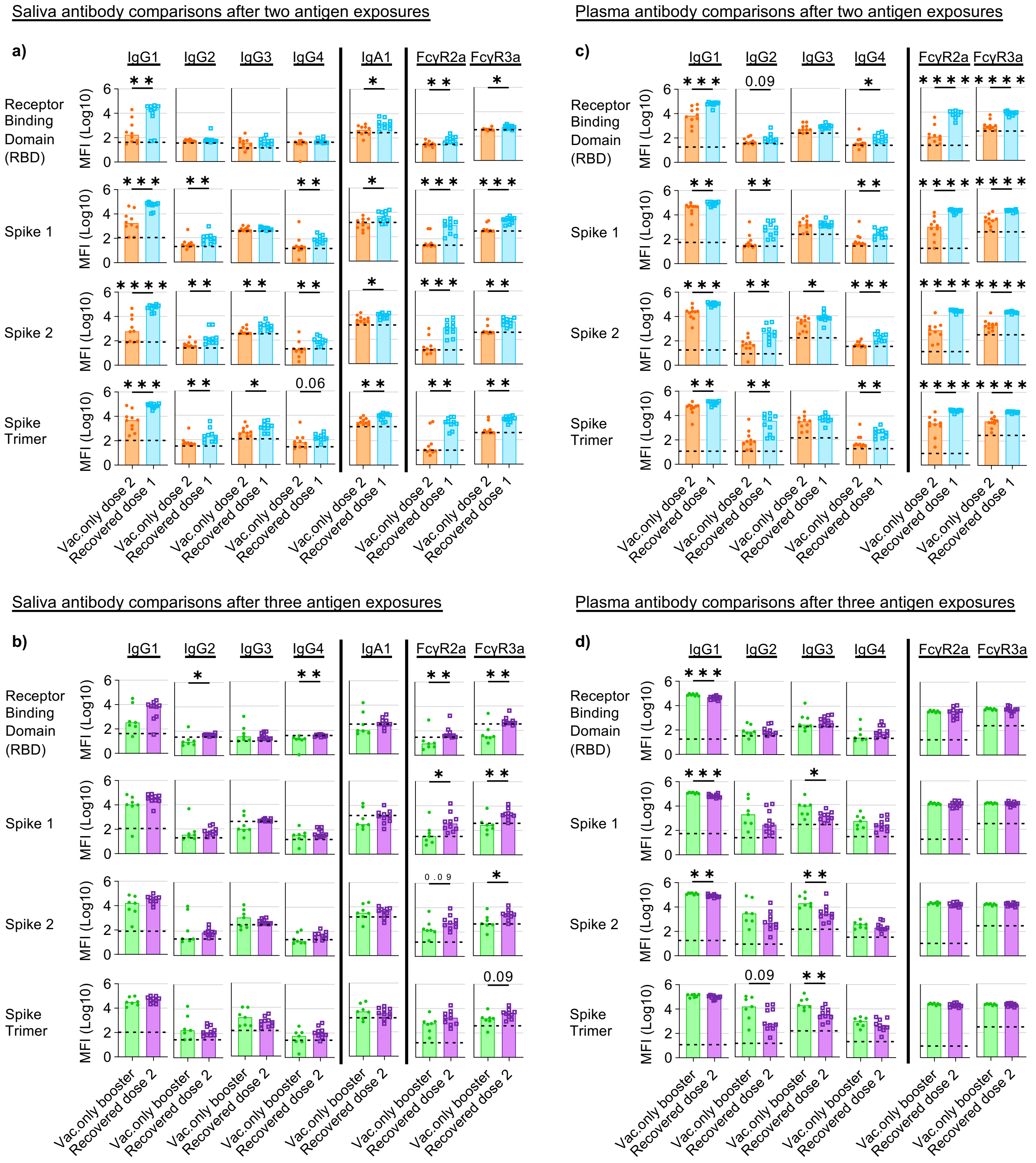

Supplementary figure 2: Comparisons of salivary and plasma antibody responses between vaccinated only and COVID-19 recovered vaccinees after 2 and 3 antigen exposures

Bar graphs show the key differences in ancestral SARS-CoV-2 spike-specific salivary **(a, b)** and plasma **(c, d)** antibody responses between vaccinated only and COVID-19 recovered cohorts after two **(a, c)** and three **(b, d)** antigens exposures. Background responses from healthy COVID-19 uninfected and unvaccinated controls are displayed by dotted lines. Statistical significance was calculated using the two-tailed Mann-Whitney *U*-test. Where significant or trending significance, *P*-values were reported (* $P \leq 0.05$; ** $P \leq 0.01$; *** $P \leq 0.001$; **** $P \leq 0.0001$).

Titration curves for SARS-CoV-2 Spike Trimer variants array

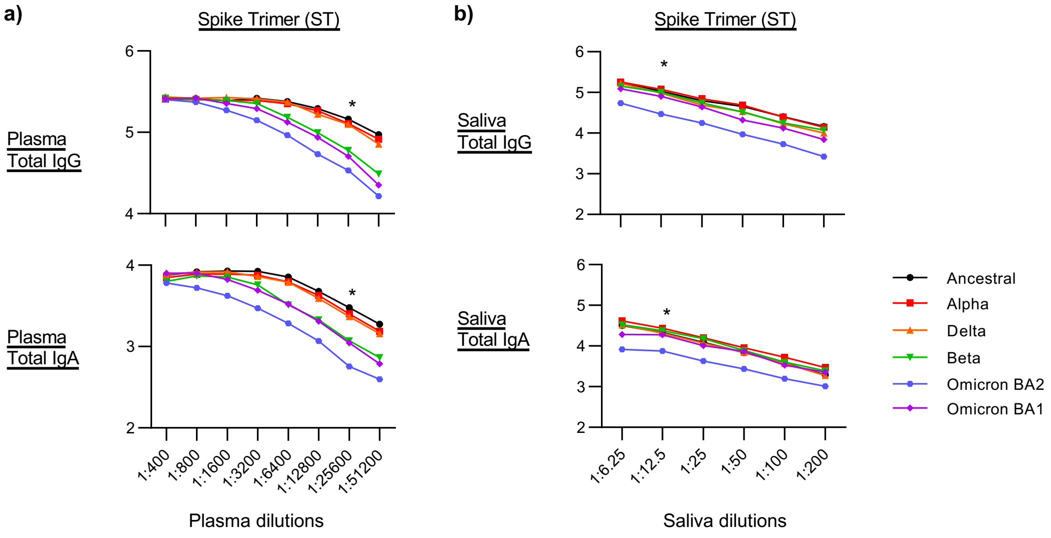

Supplementary figure 3: Plasma and saliva titration curves for SARS-CoV-2 Spike Trimer variants array

Titration curves show the plasma **(a)** and salivary **(b)** total IgG and IgA responses against ancestral WT Spike Trimer and the VoC Spike Trimers using pooled plasma from COVID-19 boostered individuals and the saliva sample from a boostered individual who seroconverted strongly during their Delta breakthrough infection. Asterisks define the respective dilutions chosen for the multiplex assays.

Saliva SARS-CoV-2 WT and VoC Spike Trimer IgA responses

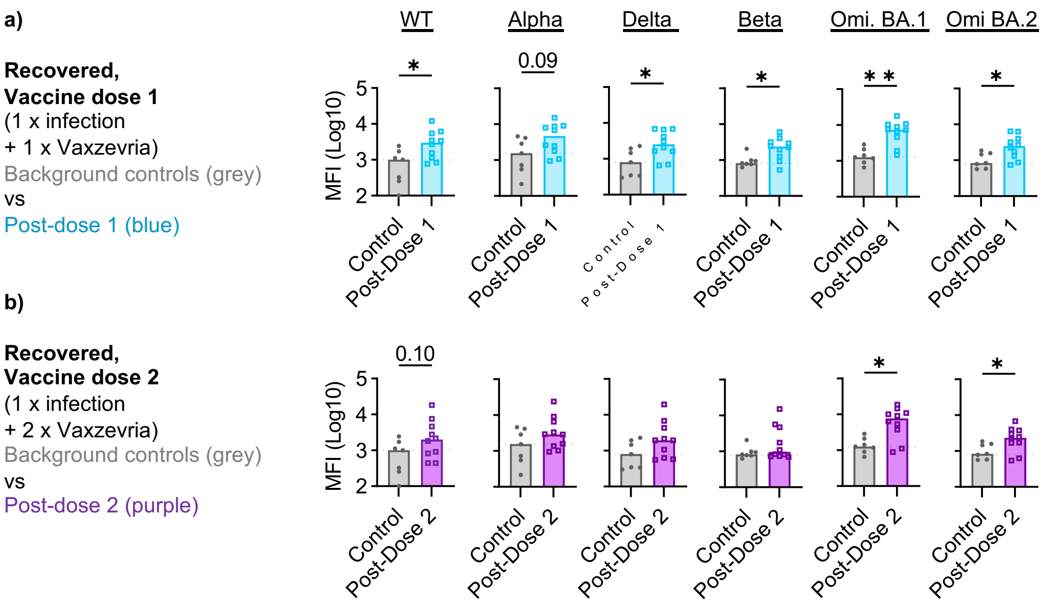

Supplementary figure 4: Saliva SARS-CoV-2 WT and VoC Spike Trimer IgA responses from healthy COVID-19 uninfected, unvaccinated background controls and Vaxzevria vaccinees

Bar graphs show the salivary IgA responses against WT SARS-CoV-2 or the VoCs from healthy COVID-19 uninfected, unvaccinated individuals (grey), as well as the salivary IgA responses from COVID-19 recovered individuals after their first **(a)** and second **(b)** Vaxzevria vaccines. Median background responses from healthy COVID-19 uninfected and unvaccinated controls are displayed by dotted lines. Statistical significance was calculated using the two-tailed Mann-Whitney *U*-test. Where significant or trending significance, *P*-values were reported (* $P \leq 0.05$; ** $P \leq 0.01$; *** $P \leq 0.001$; **** $P \leq 0.0001$).

Supplementary table 1: Cohort information for COVID-19 vaccinees (V) and COVID-19 uninfected unvaccinated healthy controls (C)

Donor:	Gender:	Age:	Time from previous COVID-19 (days):	1st vaccine dose:	Timepoint post 1st dose (days):	Time between 1st and 2nd dose:	2nd vaccine dose:	Timepoint post 2nd dose (days):	Extended timepoint post 2nd dose (days):	3rd vaccine dose:	Timepoint post 3rd dose (days):
V01	F	In their 50s	n.d.	Vaxzevria	13	85	Vaxzevria	12	n.d.	n.d.	n.d.
V02	M	In their 50s	n.d.	Vaxzevria	13	80	Vaxzevria	17	n.d.	n.d.	n.d.
V03	F	In their 60s	n.d.	Vaxzevria	22	84	Vaxzevria	14	n.d.	n.d.	n.d.
V04	M	In their 60s	n.d.	Vaxzevria	14	70	Vaxzevria	7	n.d.	n.d.	n.d.
V05	M	In their 60s	n.d.	Vaxzevria	11	84	Vaxzevria	10	n.d.	n.d.	n.d.
V06	M	In their 20s	n.d.	Vaxzevria	15	71	Vaxzevria	22	n.d.	n.d.	n.d.
V07	M	In their 50s	n.d.	Vaxzevria	13	81	Vaxzevria	n.d.	n.d.	n.d.	n.d.
V08	M	In their 60s	n.d.	Vaxzevria	7	84	Vaxzevria	7	n.d.	n.d.	n.d.
V09	M	In their 50s	n.d.	Vaxzevria	16	84	Vaxzevria	16	n.d.	n.d.	n.d.
V10	F	In their 50s	n.d.	Vaxzevria	8	84	Vaxzevria	15	n.d.	n.d.	n.d.
V11	F	In their 20s	n.d.	Vaxzevria	15	85	Vaxzevria	14	168	mRNA-1273	14
V12	F	In their 30s	n.d.	Vaxzevria	n.d.	81	Vaxzevria	n.d.	101	BNT162b2	14
V13	F	In their 20s	n.d.	Vaxzevria	n.d.	84	Vaxzevria	n.d.	124	BNT162b2	10
V14	F	In their 20s	n.d.	Vaxzevria	n.d.	84	Vaxzevria	n.d.	134	BNT162b2	14
V15	F	In their 50s	n.d.	Vaxzevria	n.d.	91	Vaxzevria	n.d.	158	BNT162b2	29
V16	F	In their 30s	n.d.	Vaxzevria	n.d.	90	Vaxzevria	n.d.	178	BNT162b2	29
V17	F	In their 20s	n.d.	Vaxzevria	n.d.	92	Vaxzevria	n.d.	184	BNT162b2	31
V18	M	In their 70s	458	Vaxzevria	15	76	Vaxzevria	16	n.d.	n.d.	n.d.
V19	F	In their 60s	427	Vaxzevria	16	84	Vaxzevria	23	n.d.	n.d.	n.d.
V20	F	In their 60s	435	Vaxzevria	8	85	Vaxzevria	14	n.d.	n.d.	n.d.
V21	M	In their 60s	435	Vaxzevria	8	84	Vaxzevria	15	n.d.	n.d.	n.d.
V22	M	In their 60s	432	Vaxzevria	15	86	Vaxzevria	12	n.d.	n.d.	n.d.
V23	F	In their 60s	432	Vaxzevria	15	84	Vaxzevria	14	n.d.	n.d.	n.d.
V24	M	In their 60s	422	Vaxzevria	13	70	Vaxzevria	14	n.d.	n.d.	n.d.
V25	M	In their 60s	418	Vaxzevria	13	77	Vaxzevria	26	n.d.	n.d.	n.d.
V26	M	In their 50s	451	Vaxzevria	12	84	Vaxzevria	18	n.d.	n.d.	n.d.
V27	M	In their 50s	398	Vaxzevria	n.d.	84	Vaxzevria	19	n.d.	n.d.	n.d.
V28	F	In their 50s	398	Vaxzevria	n.d.	84	Vaxzevria	19	n.d.	n.d.	n.d.
V29	M	In their 50s	262	Vaxzevria	12	70	Vaxzevria	21	n.d.	n.d.	n.d.
C01	F	In their 30s	n.d.	n.d.	n.d.	n.d.	n.d.	n.d.	n.d.	n.d.	n.d.
C02	F	In their 20s	n.d.	n.d.	n.d.	n.d.	n.d.	n.d.	n.d.	n.d.	n.d.
C03	F	In their 30s	n.d.	n.d.	n.d.	n.d.	n.d.	n.d.	n.d.	n.d.	n.d.
C04	M	In their 20s	n.d.	n.d.	n.d.	n.d.	n.d.	n.d.	n.d.	n.d.	n.d.
C05	F	In their 30s	n.d.	n.d.	n.d.	n.d.	n.d.	n.d.	n.d.	n.d.	n.d.
C06	F	In their 20s	n.d.	n.d.	n.d.	n.d.	n.d.	n.d.	n.d.	n.d.	n.d.
C07	F	In their 20s	n.d.	n.d.	n.d.	n.d.	n.d.	n.d.	n.d.	n.d.	n.d.
C08	F	In their 20s	n.d.	n.d.	n.d.	n.d.	n.d.	n.d.	n.d.	n.d.	n.d.
C09	F	In their 30s	n.d.	n.d.	n.d.	n.d.	n.d.	n.d.	n.d.	n.d.	n.d.
C10	F	In their 30s	n.d.	n.d.	n.d.	n.d.	n.d.	n.d.	n.d.	n.d.	n.d.
C11	M	In their 30s	n.d.	n.d.	n.d.	n.d.	n.d.	n.d.	n.d.	n.d.	n.d.
C12	F	In their 20s	n.d.	n.d.	n.d.	n.d.	n.d.	n.d.	n.d.	n.d.	n.d.
C14	F	In their 30s	n.d.	n.d.	n.d.	n.d.	n.d.	n.d.	n.d.	n.d.	n.d.
C15	F	In their 30s	n.d.	n.d.	n.d.	n.d.	n.d.	n.d.	n.d.	n.d.	n.d.
C18	M	In their 30s	n.d.	n.d.	n.d.	n.d.	n.d.	n.d.	n.d.	n.d.	n.d.
